# Guillain-Barré syndrome associated with COVID-19 infection

**DOI:** 10.11604/pamj.supp.2020.35.2.25003

**Published:** 2020-07-18

**Authors:** Hussein Karim Manji, Upendo George, Namala Patrick Mkopi, Karim Premji Manji

**Affiliations:** 1 Muhimbili University of Health and Allied Sciences, Department of Emergency Medicine, P.O. Box 65001, Dar-es-Salaam, Tanzania,; 2 Muhimbili National Hospital, Department of Emergency Medicine, P.O. Box 65000, Dar-es-Salaam, Tanzania,; 3 Muhimbili National Hospital, Department of Pediatrics, P.O. Box 65000, Dar-es-Salaam, Tanzania,; 4 Muhimbili University of Health and Allied Sciences, Department of Pediatrics, P.O. Box 65001, Dar-es-Salaam, Tanzania

**Keywords:** COVID-19, GBS, pediatrics, Guillain-Barré, SARS-CoV-2

## Abstract

We are reporting a case of Acute Post-Infectious Flaccid paralysis also commonly known as Guillain-Barré Syndrome (GBS) in a patient with confirmed COVID-19 infection. GBS often occurs following an infectious trigger which induces autoimmune reaction causing damage to peripheral nerves. So far, only 8 cases have been described in association with COVID-19. This is the first to be described in Tanzania in an African Child, and probably the first in the continent. This report is presented for clinicians to be aware and for the medical fraternity to look into this unusual presentation which may shed some more light on possible pathways of the pathogenesis and clinical manifestations. We recommend that the presentation of GBS with acute respiratory distress should warrant extra precaution and a testing for COVID-19 especially when the symptoms of COVID-19 are protean.

## Introduction

A novel Corona virus termed COVID-19 was first reported in Wuhan, China as early as December 2019 [1]. The first known case reported in Tanzania was on the 16th of March 2020 in Arusha, Tanzania [2]. Intense research and surveillance was started worldwide after the detection of this virus and its potential for spread. The first reported death in Tanzania from COVID-19 was reported on 31st March 2020. Although much is known about the mortality associated with this virus, there is a paucity of information related to its pathophysiology. It has been known to gain entry into cells by fusing with the angiotensin-converting enzyme 2 receptor and inducing inflammatory reactions with progression from the upper respiratory tract down to the lungs with resultant hypoxia, ground glass infiltrates and progression to Acute Respiratory Distress Syndrome [ARDS] [3,4]. The presentations of COVID 19 have ranged from asymptomatic cases and those with mild symptoms to severe cases associated with high mortality. The common symptoms implicated after a mean incubation period of 5.1 days include cough and shortness of breath or difficulty breathing associated with fever, chills, myalgia, headache, sore throat and/or new loss of taste or smell with other nonspecific reported symptoms including fatigue, diarrhea and malaise [5,6]. Although there was scant data on COVID 19 in children, Dong et al. presented intriguing findings in his study investigating >2000 children with suspected or confirmed COVID 19 [7]. Children are less likely to become severely ill compared to older adults, although those with underlying pulmonary pathologies, immunosuppressed states and younger age are more at risk. While numerous studies continue to be conducted on the various presentations and complications of COVID 19 in the world, it is important to identify the serious neurological sequelae associated with this viral infection which often go unnoticed [8].

Mao et al. reported 36.4% of the 214 study participants hospitalized with COVID 19 had neurological manifestations [9]. In our knowledge, there have been seven cases of Guillain-Barré syndrome [GBS] reported so far with five in Italy, one in Iran and one in the United States in patients with COVID 19 infection. In Italy, the first symptoms noted were lower limb weakness and paresthesia in four patients and facial diplegia followed by ataxia in one patient with symptoms of GBS occurring within 5-10 days from first onset of symptoms of COVID 19 [10]. Virani et al. reported a SARS-CoV-2 positive patient who developed progressive, ascending weakness consistent with GBS [11] while in Iran is where the first case was reported describing symptoms of GBS in an infected patient with COVID 19 [12]. GBS is an acute immune mediated demyelinating disease which is progressive, ascending, symmetrical and involves flaccid paralysis of the limbs with areflexia or hyporeflexia with or without cranial nerve involvement with history of preceding respiratory or gastrointestinal infection 2-4 weeks prior in two third of cases [13]. In this report, we describe GBS symptoms in a 12 year old infected patient with COVID-19 for the first time in Tanzania and Africa as a whole.

## Patients and Observation

LB, 12 year old young man presented to the Emergency Department with symptoms of acute progressive symmetric ascending quadriparesis with bilateral facial paresis. He was asymptomatic a week prior and then he developed a low grade fever and a dry cough. He was managed conservatively and treated symptomatically. He complained of lower back pain 5 days prior to arrival to the ED which was rapidly followed by weakness of both lower limbs. He was able to walk initially and was taken to a local dispensary where he was administered an intramuscular analgesics and discharged. The next morning, he lost function of both lower limbs with associated weakness of bilateral upper limbs as well and could not get out of bed and this prompted the family to seek further medical attention. He was taken to the same facility again and advised to start physiotherapy with a provisional diagnosis of lumbar spondylitis. He was referred when he developed facial paresis bilaterally and an altered level of consciousness associated with an increased respiratory rate. In the emergency department, the mother provided the additional information of the low grade fever and cough from the week before which they had managed conservatively at home. There was no history of recent travel or contact with a positive case of COVID 19. There was no significant past history or any known co-morbidities.

**Figure 1:**
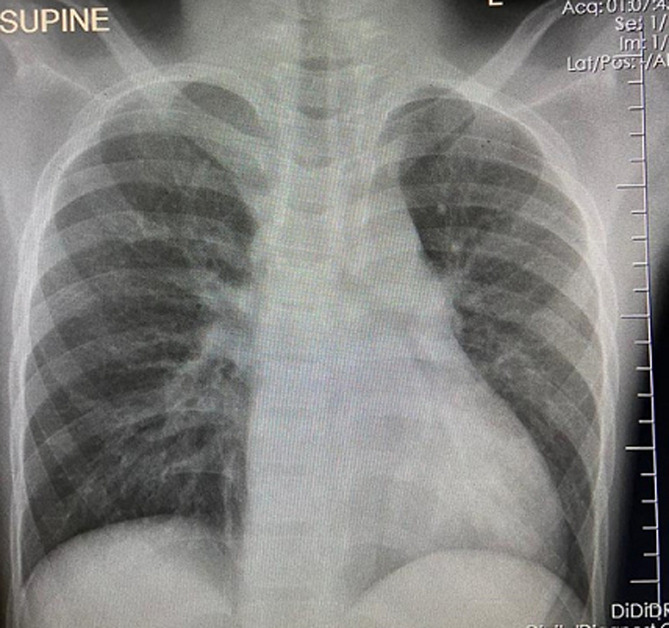
chest radiograph of the patient

Vital signs on presentation were remarkable with a respiratory rate of 24 breaths per minute with oxygen saturation of 88% [hypoxic] on room air with a heart rate of 150 beats per minute and a blood pressure of 150/100 mmHg. The patient was barely conscious with a Glasgow Coma score of 6/15 and had signs of shock with cold extremities and diaphoresis. The child was placed on nasal prongs at 6L of high flow oxygen and connected to a cardiac monitor. Blood was drawn for lab investigations, an ECG was done and a chest X-ray ordered. Physical examination revealed decreased lower extremity strength and muscle tone compared to upper extremities with a score of 1/5 in lower extremities versus a score of 2/5 in upper limbs as per Medical Research Council [MRC] scale. Deep tendon reflexes were absent in all four limbs. Meningeal irritation signs and upper motor neuron disorder signswere absent. The bedside blood glucose was 11.0 mmol/L. His lab results showed a white blood cell count of 17.3 x103 cells/mL, hemoglobin of 14.6g/dL and platelet count of 361 x 103 cells/mL with an otherwise normal differential. Electrolytes and renal profile were within normal ranges with a blood urea nitrogen of 5.6 mmol/L, creatinine of 54.4µmol/L, sodium of 142, potassium of 4.6 and chloride 100. Chest X-ray revealed bilateral basilar opacifications suggestive of consolidation with diffuse infiltrates bilaterally. ECG showed a sinus arrhythmia with slightly tall and tented T waves (Figure 1). Bedside ultrasound revealed good cardiac contractility with a full Inferior vena Cava [IVC] and no pericardial or pleural effusion.

Given the existing epidemiological scenario and preceding symptoms of fever and cough, COVID-19 infection was suspected. Appropriate isolation precautions were implemented, and a respiratory viral panel testing [nasopharyngeal PCR] was sent. Antibiotic coverage was done empirically and other treatment modalities done as required. Just prior to transfer to Pediatric Intensive Care [PICU], child lost pulse and Cardio-Pulmonary Resuscitation [CPR] was initiated with return of spontaneous circulation [ROSC] achieved within 1 cycle of the CPR. Child was intubated and ventilated and started on positive inotrope infusion. A diagnosis of GBS was made and patient was immediately started on 400mg/kg of intravenous immune globulin [IVIG] therapy for a planned 5-day course according to clinical manifestations related to GBS. The reverse transcription-polymerase chain reaction [RT-PCR] for COVID-19 was positive and further evaluation and management was continued in the ICU. Given the classic clinical picture of GBS in absence of other identifiable etiology for his neurologic disease and strict infection control measures, additional supportive testing was not pursued. His clinical course showed improvement in his respiratory status, neurological status as well as his consciousness level with an improvement of the GCS to 14/15 within 5 days. Neurologically, his upper extremity weakness resolved after completion of the course of IVIG. Lower extremity power and tone improved significantly. He was planned for weaning off ventilation and extubation on day 6, and eventual rehabilitation and discharge. During the night preceding the planned weaning, the child self extubated accidentally and rapidly decompensated. Cardiac arrest ensued and attempts to resuscitate after CPR were futile and child died.

## Discussion

Despite having first been reported in Wuhan, China; at the time of preparing this paper, the SARS-CoV-2 has infected over 3.2 million people worldwide with over 229,000 deaths attributed to COVID-19 directly [14]. In Tanzania alone, 480 cases have been reported so far with 16 reported cases, not taking into consideration the possible missed cases and deaths and those not reported. In this study, we reported Guillain Barré syndrome in a patient infected with COVID-19. The major causes of death in COVID-19 patients are acute respiratory distress syndrome [ARDS] and the resultant hypoxia as well as the cytokine storm causing an overwhelming shock. Our patient had signs of shock despite no volume depletion or cardiogenic issue prompting another cause, either septic, neurogenic or of this obvious COVID-19 pathology. GBS is an acute progressive symmetrical demyelinating disease whose symptoms typically develop 3 days to 6 weeks after an upper respiratory infection or a diarrheal disease [15].

Although classically associated with Campylobacter jejuni, viruses like EBV, CMV, HIV and recently even Zika virus have also been implicated in some cases. The pathophysiology suggested is an autoimmune reaction secondary to molecular mimicry between the surface glycoproteins of the offending pathogen and the structures on peripheral nerves causing the antibodies to attack the nerves and cause neurologic symptom [13,15]. We believe the offending agent in our case which triggered this neurological process was COVID 19 which also caused the respiratory symptoms. There is no direct evidence of a causative relationship between GBS and COVID 19 but due to various reports of similar cases; inclusive of the 5 cases reported in Italy [10], the one case in Iran [12] and the case in the US [11]; all from different epidemiological and clinical settings indicates a common factor which raises the index of suspicion of the pandemic virus being the possible trigger. Moreover, previous research has implicated other corona viruses in neurologic disease. Human multiple sclerosis was modeled in an immunocompromised mouse infected with a corona virus variant by Houtman and Fleming [16,17].

Also evidence has shown spike glycoprotein involvement with murine infection of the neurologic system with support from the hemagglutination proteins, making betacoronavirus a more likely strain of corona virus to infect neurologic cell lines [18]. Neuromuscular involvement was also reported in patients infected with the SARS-CoV-2 [19]. This case also highlights the need for guarded sedation and probably early weaning to prevent a conscious young man from inadvertent extubation. To date, there have been very few reported cases of GBS associated with SARS-Cov-2 infection. The authors of these reported cases suggested a possible association between the GBS and COVID-19 pending more epidemiological data. Our case adds to these recently reported cases. Moreover, the presentation in our patient suggests the ‘para-infectious’ profile rather than the generally encountered relatively longer time period between the triggering event and development of GBS.

## Conclusion

It is known that GBS is often triggered by a preceding bacterial or viral infection and hence COVID 19 must be considered a trigger to rule out by clinicians in such a case as the GBS maybe a neurological complication of the viral illness. Also, it raises the index of suspicion in patients whose presenting symptoms are neurological, especially when acute, and a thorough and detailed review of all systems is crucial to identify other features possibly suggestive of COVID-19. It is important to identify the GBS and start IVIG early for optimal outcomes. This report also adds stress to the fact that not all cases of COVID 19 will present with typical features or vital signs on presentation to the ED. Lastly, appropriate infection prevention measures and personal protective equipment are crucial in any and every case and in approaching any patient coming to the emergency department due to the variation in presentation of patients with COVID 19.
